# Infant siblings and the investigation of autism risk factors

**DOI:** 10.1186/1866-1955-4-7

**Published:** 2012-04-18

**Authors:** Craig J Newschaffer, Lisa A Croen, M Daniele Fallin, Irva Hertz-Picciotto, Danh V Nguyen, Nora L Lee, Carmen A Berry, Homayoon Farzadegan, H Nicole Hess, Rebecca J Landa, Susan E Levy, Maria L Massolo, Stacey C Meyerer, Sandra M Mohammed, McKenzie C Oliver, Sally Ozonoff, Juhi Pandey, Adam Schroeder, Kristine M Shedd-Wise

**Affiliations:** 1 Department of Epidemiology and Biostatistics, Drexel School of Public Health, 1505 Race Street, Mail Stop 1033, Philadelphia, PA 19102, USA; 2 Kaiser Permanente Division of Research, 2000 Broadway, Oakland, CA 94612, USA; 3 Department of Epidemiology, Johns Hopkins Bloomberg School of Public Health, 615 N Wolfe Street, Baltimore, MD 21205, USA; 4 Department of Public Health Sciences, University of California, Davis, CA 95616, USA; 5 Kaiser Permanente San Jose Medical Center, 6620 Via Del Oro, San Jose, CA 95119, USA; 6 Kennedy Krieger Institute, 3901 Greenspring Avenue, 2nd Floor, Baltimore, MD 21211, USA; 7 Center for Autism Research, The Children's Hospital of Philadelphia, 3535 Market Street, Suite 860, Philadelphia, PA 19104, USA; 8 The MIND Institute, UC Davis Medical Center, 2825 50th Street, Sacramento, CA 95817, USA

**Keywords:** Autism, Cohort, Epidemiology, Pregnancy, Prospective, Sibling, Study Design

## Abstract

Infant sibling studies have been at the vanguard of autism spectrum disorders (ASD) research over the past decade, providing important new knowledge about the earliest emerging signs of ASD and expanding our understanding of the developmental course of this complex disorder. Studies focused on siblings of children with ASD also have unrealized potential for contributing to ASD etiologic research. Moving targeted time of enrollment back from infancy toward conception creates tremendous opportunities for optimally studying risk factors and risk biomarkers during the pre-, peri- and neonatal periods. By doing so, a traditional sibling study, which already incorporates close developmental follow-up of at-risk infants through the third year of life, is essentially reconfigured as an enriched-risk pregnancy cohort study. This review considers the enriched-risk pregnancy cohort approach of studying infant siblings in the context of current thinking on ASD etiologic mechanisms. It then discusses the key features of this approach and provides a description of the design and implementation strategy of one major ASD enriched-risk pregnancy cohort study: the Early Autism Risk Longitudinal Investigation (EARLI).

## Review

### Introduction

In 1957, Pearson and Kley published a prescient paper asserting that neuropsychiatric research should capitalize on the "tendency of particular abnormalities of behavior to run in families" (p. 406) so that "subpopulations defined in terms of genetic relationship to index cases...might be studied longitudinally..." (p. 418) [1]. They went on to note that such studies could be effective and economical for etiologic research. A 1976 review of the genetics of infantile autism and childhood schizophrenia [2] highlighted the potential of Pearson and Kley's high-risk design for etiologic research, but at that time the only such studies underway were investigations focusing on children of parents with schizophrenia (reviewed by Garmezy [3]). In the 1980s, prompted by the 1977 publication of Folstein and Rutter's seminal autism twin study [4], siblings of autism probands increasingly were included in research samples; however, these were largely cross-sectional family studies in which researchers looked at recurrence risk and genetic segregation or linkage, not at prospective investigations where at-risk siblings were the subjects of principal interest. The first consideration of the prospective infant sibling study in autism, according to Yirmiya and Ozonoff, occurred in the mid-1980s, when US and UK researchers contemplated but rejected the idea because of concerns over heterogeneity in index proband diagnosis [5]. Once standard diagnostic tools were developed in the early 1990s, these projects moved forward with a focus firmly on phenotypic antecedents and very early signs of autism spectrum disorders (ASDs). Rogers [6] has since described the discovery of "the first behavioral characteristics that predict development of autism" as the "Holy Grail" (p. 126) of autism infant siblings research. Today there are 25 infant sibling research teams that are part of the High Risk Baby Siblings Research Consortium (BSRC) (Autism Speaks, Research on High Risk Baby Sibs: http://www.autismspeaks.org/science/initiatives/high-risk-baby-sibs), a voluntary network of projects with funding support from the National Institutes of Health and Autism Speaks, united in their common purpose of pursuing early phenotypic predictors of autism. The first papers derived from these efforts began to appear in 2005 and 2006 [7-10], and findings on emergence and trajectory of a range of developmental outcomes in high-risk infant sibling cohorts have been published steadily in the literature ever since.

Although discovering robust early phenotypic markers for autism to facilitate early detection and intervention remains a major autism research goal and an appropriate priority for infant sibling studies, the application of the high-risk infant sibling study design in autism etiology research has been underexplored. In this paper, we consider the role that infant sibling designs can play in autism risk factor research in the context of the evolving understanding of autism etiology and describe the design and methods which are being employed by a major high-risk sibling cohort study focused on autism etiology: the Early Autism Risk Longitudinal Investigation (EARLI).

### Current thinking on etiologic mechanisms in autism

For decades, multiple lines of evidence have supported a substantial heritable component of autism etiology, including twin studies [4,11-14], familial risk studies [15-24], segregation analyses [24-27] and reported correlations between autism phenotypes and other congenital genetic disorders [28-32]. Modern genomic methods applied extensively to a variety of autism samples over the past decade have underscored the complexity of autism inheritance. A number of rare variants, for the most part *de novo *or inherited copy number variations (CNVs) [33-35], have been linked to autism by virtue of their apparent high penetrance. The teams leading three major autism genomewide association studies (GWASs) [33,36,37] have generated additional candidate genes, but have failed to replicate each other's findings. Consequently, lists of plausible autism candidate genes now include well over 100 genes [38,39], including common genetic variants likely to have very small independent effects but potentially contributing to mechanisms with larger effects by interacting with each other or with rare genetic events [40]. Therefore, ongoing efforts are focused on the use of sophisticated analytic techniques applied to genomic data to identify common, biologically plausible pathways along which gene-gene interactions may take place [41-44].

In addition to an emphasis on gene-gene interactions, nearly all recent comprehensive reviews of autism genetics have cited interplay between genetic mechanisms and environmental exposures as another plausible contributor to the complexity of autism etiology [45-52]. The recent twin study conducted by Hallmayer *et al. *[53], which was larger than all its predecessors and the first done with autism cases confirmed using today's diagnostic tools, suggested a far larger role for environmental factors than did any earlier twin study. Furthermore, the fact that dizygotic twin concordance in their study was substantially larger than nontwin sibling recurrence risk reported in a recent large study of infant siblings [54] points to the prenatal period specifically as a period of special interest with respect to environmental influences. A potential role for epigenetic mechanisms in autism etiology [55,56] also suggests additional ways in which environmental exposures can work in concert with genomic factors [57]. The need to move forward with more extensive investigation of environmental risk factors in autism is now widely accepted.

For most of the past 40 years, the investigation of environmental exposures has been sporadic. Several studies have provided evidence of highly elevated risk arising from congenital exposure to rubella [58,59] and cytomegalovirus [60]. Similarly, some pharmacologic exposures in the prenatal period have been linked to autism, including thalidomide [61] and valproic acid [62-64]. More recent epidemiologic research has underscored the prenatal period as the most relevant etiologic window for autism environmental risk factors. For example, large studies have continued to find associations of autism risk with prenatal medication use [65,66] and infection [67]. Consistent with the infection finding, investigators in a small case-control study who capitalized on banked midpregnancy blood samples reported more frequent elevations in certain circulating inflammatory cytokines in mothers of children with autism than controls [68]. The first system-level analysis of the ASD brain transcriptome, in addition to an expected finding of synaptic dysfunction, has also suggested the presence of immune dysregulation [69], which is consistent with an earlier finding of neuroinflammation in the brains of individuals with autism [70]. However, whether these indications derived from autopsy studies reflect antecedent and potentially causal immune-mediated events or downstream responses to other autism neuropathology is not yet clear.

Recently published systematic reviews of traditional obstetric and neonatal risk factors and autism reported that, for most of the individual risk factors considered (for example, contraception prior to pregnancy, maternal obstetric history, bleeding in pregnancy, gestational diabetes), either insufficient data were available or findings have not been well-replicated in the published literature [71-73]. This is not entirely unexpected, as many past studies have been based on small clinical samples without confirmation of diagnoses. However, the one factor with the most consistent association with increased autism risk across multiple studies is advanced parental age [74-80]. A variety of mechanisms might explain these associations, such as increased maternal complications during pregnancy or delivery, an accumulation of toxins affecting either the intrauterine environment or sperm development, and induced *de novo *mutation, of particular interest in the case of older fathers.

An interesting obstetric risk factor examined only recently with respect to autism is interpregnancy interval. An interval of less than one year between pregnancies was found in an initial report to be associated with more than a threefold increase in autism risk compared to intervals of three or more years (OR = 3.4, 95% CI = 3.00 to 3.82). If short interpregnancy interval is an autism risk factor, it could implicate the intrauterine environment through nutritional depletion mechanisms [81]. Indeed, researchers in a large case-control investigation have reported intake of prenatal vitamin supplements in the periconception period (three months prior and one month after conception) to confer nearly a 40% reduction in risk (OR = 0.62, 95% CI = 0.42 to 0.93) [82]. This study was also notable because it contains the only published results to date explicitly supporting a gene-environment interaction in autism with the apparent protection from maternal prenatal vitamin use magnified in the presence of certain genotypes involved in one-carbon metabolism [82].

Several investigations have examined air pollution, a complex mixture of exposures with wide-ranging toxicities, in relation to autism diagnoses. The designs of these ranged from a purely ecologic design that focused on industrial emissions of a single pollutant [83], to investigations that utilized individual-level diagnostic information in relation to modeled estimates of 25 hazardous air pollutants [84,85], to distance to freeway, a strong indicator of ambient traffic-related pollutant levels [86]. This most recent study, a case-control design using clinically confirmed cases and individual-level exposure information, found living within one-quarter mile of a freeway at the time of delivery was associated with a 1.9-fold increased ASD risk (95% CI = 1.04 to 3.45). Researchers in earlier studies had used exposures occurring in the second year of life or later, which might not be the most etiologically relevant period.

Investigators in a number of other studies have also explored potential associations between autism diagnosis or autism-related phenotypes and pesticide exposure in the prenatal period. Residence in a location where application of organochlorine pesticides reached levels falling into the highest nonzero quartile during the eight-week pregnancy period after closure of the cranial neural tube was associated with a sixfold higher odds ratio of the child's developing autism (OR = 6.1, 95% CI = 2.4 to 15.3) [87]. In a cohort study of primarily Mexican-American women, higher levels of metabolites for organophosphate pesticides were found to predict higher scores on an autism-related scale in 24-month-olds [88]. Studies attempting to replicate these findings are needed, though both types of compounds are plausibly linked to altered central nervous system development through endocrine disruption for the long-lasting organochlorines and through direct toxicity to the developing brain for the rapidly cleared organophosphates. Other commonly used pesticides have been associated with general neurodevelopmental deficits in prospective pregnancy cohorts [89-91] but have yet to be studied in autism.

With epidemiologic evidence consistently pointing to the prenatal period as a window of vulnerability to environmental exposures in autism, one might ask whether this is consistent with known autism neuropathology. Indeed, pathologic changes documented in autopsied brains of individuals with autism, including those found in the brainstem [92], cerebellum [93,94] and cortex [95], are indicative of a pathologic process originating *in utero*. Early brain overgrowth in autism, now documented in two longitudinal brain imaging studies [96,97], also suggests the presence of causal events occurring prior to birth, as does the recent brain transcriptomics report that autism brains lacked a pattern of differential gene expression across frontal and temporal cortical regions [69] that typically emerges during fetal development [98].

Could prenatal causal events be linked to exogenous exposures? It has long been established that prenatal brain development, including the fundamental processes of neuronal proliferation, migration, differentiation, synaptogenesis, gliogenesis, myelination and apoptosis, are susceptible to disruption by environmental exposures [99,100]. Subsequently, each of these fundamental processes has been considered in alternative models of autism pathology [101]. Some of the more recent work geared toward using autism genomics to identify biologic pathways has implicated synaptic homeostasis as a candidate common biological process in autism [102]. Although synapse formation begins in the third trimester, with synapse restructuring and connectivity development continuing well into postnatal life, animal models have shown that environmental exposures earlier in pregnancy can lead to impaired postnatal synaptic activity without obvious signs of disruption prenatally [103]. In addition, other genomics efforts focused on pathway detection, one using GWAS data and the other CNV data, have independently implicated impaired neuronal projection and axonal guidance [35,44] as mechanisms of chief interest. These are environmentally sensitive processes beginning early in brain development [104,105] that could certainly affect synaptic functioning downstream. Of course, the high likelihood of an *in utero *origin of autism in no way rules out the potential for etiologic and prognostic influences after birth, but the design and implementation of etiologic research focused on the prenatal period would appear strongly justified.

### Expanding the infant siblings approach to study autism etiology

Infant sibling cohort studies, as implemented by members of the BSRC, enroll subjects younger than 18 months of age (many as young as 6 months of age) and carry out close longitudinal developmental follow-up, typically through 3 years of age. The design choice was motivated in part by expected recurrence rates that were many times higher than population autism prevalence and in part by the opportunity to observe early behavioral markers and better understand the complex early natural history of ASDs afforded by carefully measuring development prospectively. Both of these considerations are also quite germane to etiologic research. Yet, to maximally capitalize on infant sibling designs for etiologic research, it is necessary to extend cohort enrollment back to a point where the mother and the developing fetus can be followed prospectively through windows of potential etiologic vulnerability; in other words, by transforming the design to a high-risk pregnancy cohort. Each of these three features, increased event rate, prospective developmental assessment and shift to a pregnancy cohort design, is each discussed further below.

#### Increased event rates

When the BSRC was formed, published estimates for sibling recurrence risk ranged from 2% to 9% [13,15-22,24,106]. Almost all of these studies considered recurrence of the more narrow autistic disorder diagnosis among siblings of a proband with autistic disorder, although the one study of probands and siblings with any autism spectrum diagnosis reported recurrence within the same range (5.3%) [106]. In 2011, the BSRC published their first findings on ASD recurrence among 684 siblings of probands with an ASD followed from at least 18 months until at least 36 months [54]. In this large, recently ascertained sample, recurrence was 18.7% (95% CI = 13.3% to 25.5%). Even with recent population ASD prevalence estimates approaching 1% [107], this implied 20-fold increase in sibling risk translates into increased numbers of cases in an enriched-risk sibling cohort, which increases power to detect associations between risk factors and ASD case status. In addition, the presence of higher levels of subthreshold impairment in toddler-age siblings of ASD probands has been documented. For example, Toth *et al. *[108] found significant differences in expressive and receptive language, composite IQ, adaptive behavior and social communication skills when they compared (1) 42 toddler-age siblings of ASD probands who did not meet ASD criteria based on the toddler-version Autism Diagnostic Interview-Revised (ADI-R) [109] and the Autism Diagnostic Observation Schedule (ADOS) [110] to (2) 20 typically developing toddlers with no ASD family history. This suggests that there is a considerable range of impairment in the infant sibling cohort which should translate into increased power for risk factor analyses using dimensional as opposed to categorical phenotypic outcomes. Last, for conditions such as ASD where complex genetic mechanisms underlie increased baseline risk in the infant sibling sample, if a risk factor's effect is amplified by an unknown genotype or genotypes, the power to detect that risk factor is affected favorably [111].

#### Prospective developmental assessment

BSRC studies prospectively evaluate a range of developmental end points, including motor development, repetitive behaviors and abnormal movement patterns, social and emotional development, and response to joint attention [6]. The prospective developmental assessment in infant sibling studies can support etiologic research in two ways.

First, it allows for careful characterization of autism-related dimensional phenotypes at early ages. As mentioned above, dimensional end points may prove revealing in ASD etiologic research, and perhaps studying variance of traits expressed very early in life could be the most revealing. To date, the dimensional measures used in etiologic research have been those developed from assessment of older children [112-114]. Should measures that are now being used in BSRC studies such as the Autism Observation Scale in Infants (AOSI) [115] provide valid early measurement of quantitative traits related to autism, enriched risk pregnancy cohort studies could incorporate these and utilize them as continuous end points in risk factor analyses.

Second, the longitudinal characterization of development could lead to the identification of distinct developmental trajectories which might themselves be considered as outcomes or could be used to stratify cases to test hypotheses that cases with different developmental trajectories could have distinct sets of risk factors. Landa and Garrett-Mayer [10] have already examined trajectories within high-risk siblings on a range of items measured by the Mullen Scales of Early Learning [116,117] among those meeting or not meeting research criteria for ASD at 24 months and found generally flatter trajectories in the group meeting these criteria, although they noted different patterns in different domains, such as the ASD group's deviating at 14 months on fine motor performance. Rozga *et al. *[118] reported no differences at six months of age in joint attention and requesting behaviors between high-risk siblings who went on to meet criteria for ASD and those who did not, but they found an emergence of differences at age 12 months. Another recent report, however, found head lag at 6 months of age to be predictive of social and communication impairment in high-risk siblings at age 36 months [119]. As the size of infant sibling cohorts grows, BSRC investigative teams will be able to employ more sophisticated analyses to identify unique developmental trajectories, both within and across groups defined by whether ASD criteria are met.

#### Shift to a pregnancy cohort design

The returns from expanding infant sibling research can be amplified with the shift to a pregnancy cohort design. This approach allows for the prospective collection of detailed risk factor data during the critical etiologic windows, as opposed to retrospective collection that would be necessary if cohorts of siblings enrolled as toddlers were used for risk factor research. For a number of risk factors of general interest in the prenatal and neonatal periods, validation studies have demonstrated superiority of prospective versus retrospective data collection. For example, retrospective recall of depressive symptoms in pregnancy at just six months postpartum compared to prospective documentation showed only moderate agreement [120]. Recall of prenatal influenza infection symptoms (for example, persistent cough and fever) at delivery suggest underreporting compared to questionnaires completed between the 18th and 25th weeks of pregnancy [121]. Furthermore, there are concerns that parents of affected children will recall exposures during the prenatal period differently from parents of unaffected children, a phenomenon documented for certain exposures with respect to birth defects outcomes [122].

Certain findings that have begun to emerge regarding potential environmental risk factors for autism have limitations with respect to exposure measurement that could be obviated through prospective data collection in an expanded infant sibling study design. For example, one study of air pollution used modeled estimates covering the second year after birth [84]. Although the associations that were observed could contribute to the development of autism, exposures in earlier years (for example, during gestation or the first year after birth) might be of greater relevance. Other retrospective epidemiologic investigations have explicitly considered exposure during the prenatal period but have been limited to maternal residence at the time of birth as a proxy for exposure. For example, one retrospective study modeled prenatal exposure to pesticide based on distance from residence to reported date and location of agricultural applications of pesticides, but there was no individual-level measurement of exposure available [87]. The extent to which modeled exposure reflects actual exposure is a major question, with factors such as address changes, time spent at home, wind speed and drift, as well as absence of data on other exposure sources, contributing to potential misclassification. In addition, a number of studies have used administrative and medical databases to examine maternal prenatal use of medications. These data sources provide unbiased assessment for case-control comparisons and can address the relevant time periods, but they do not include data on over-the-counter medication use and do not take into account the fact that not all prescriptions are filled and not all filled prescription medications are actually taken by the patient. The infant sibling pregnancy cohort design has the potential to combine self-report data on actual use of medications with medical records documentation on prescriptions from all sources.

The shift to a prospective cohort design also creates opportunities for implementation of cost-effective analytic strategies. When, for example, laboratory assays need to be completed on stored biologic specimens to generate risk factor data, these assays can be done on select subsamples from the cohort to conserve resources. Analyses can be limited to identified cases contrasted to a sample of noncases selected from the cohort at the time of case occurrence (incidence-density matched case-control design), a sample of noncases selected at the end of follow-up (cumulative incidence case-control design) or a sample of cohort members at baseline (case-cohort design). Such designs can often achieve close to comparable statistical power to analyses of data from the entire cohort [123]. Furthermore, when data on the full cohort can be used to inform the sampling of cases and controls (for example, two-stage or countermatching designs), additional statistical efficiencies can be achieved [124,125]. Alternatively, subsamples can be selected for more resource-intensive risk factor data collection (for example, through biomarkers or medical records abstraction), and the data derived from these subsamples can be used to correct measures of association based on risk factor data available in the full cohort or in case-control samples drawn from the full cohort [126].

The current thinking on autism etiology, where causal mechanisms are believed to be complex and multifactorial, is that a role for environmental factors is quite likely. However, research on environmental risk factors for autism faces significant challenges. Critical periods may occur early in brain development, and accurate measurement of environmental exposure during these critical periods will be important if causal contributions of these factors in the context of other contributors are to be identified. Etiologic heterogeneity in autism is likely, and identification of phenotypic correlates that mark distinct etiologies or, perhaps more realistically, can serve as useful endophenotypes for identifying certain causal components, is an active area of ongoing research. Given this situation, the expansion of infant sibling study designs for etiologic research where exposure data are captured prospectively during potentially relevant critical windows, outcomes are also prospectively characterized in detail, and event rates are higher than in population-based samples would appear to be one quite useful research approach. The sections that follow provide an overview of a large multisite investigation now underway that is implementing this study design.

### The EARLI Study as a model for risk factor research using a high-risk infant sibling design

The EARLI Study was conceived to capitalize on expanding the infant siblings approach to autism etiologic research to realize many of the benefits described above. At least one other autism enriched-risk pregnancy cohort study that has many features in common with EARLI is now underway (the Markers of Autism Risk in Babies-Learning Early Signs (MARBLES) study: http://marbles.ucdavis.edu/). Below the EARLI research design, study population, recruitment and enrollment approach, risk factor data collection and outcomes evaluation strategy are described. Other investigators looking to replicate or incorporate aspects of this approach may benefit from the information provided herein. The EARLI Study parent protocol was reviewed and approved by the Drexel University Institutional Review Board (Project no. 71109; Protocol no. 17862). Local IRB approvals were also obtained at all EARLI Study sites. All study participants have undergone the consent process and signed the relevant consent or assent forms as appropriate for age and cognitive ability, or have had a parent consent on their behalf.

#### Study design

**Figure 1:**
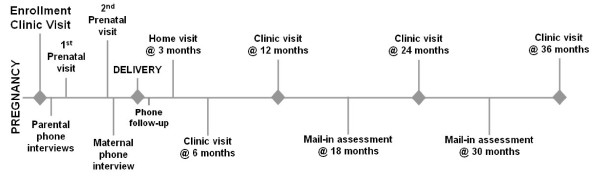
**Early Autism Risk Longitudinal Investigation Study data collection points over the course of participation**.

The EARLI Study is a multisite prospective pregnancy cohort study of mothers of children with an ASD diagnosis (autistic disorder, Asperger syndrome or pervasive developmental disorder not otherwise specified (PDD NOS)) who have become pregnant. Mothers are followed through pregnancy and delivery, then the pregnancy cohort evolves into an infant siblings cohort followed through age three years. Eligible pregnant women are enrolled by the 28th week of pregnancy, along with their biological child who has an ASD (the study proband). The biological father of the current pregnancy is also invited to enroll, although his participation is not a requirement for the participation of the mother and proband. Enrolled mothers are followed closely with intensive data and biosample collection. During the pregnancy, two to four study visits occur, which entail collection of serial biological samples from the mother and dust samples from the home. At the time of delivery, placental tissue, cord blood, heel stick blood and meconium are collected. Serial biological samples are collected postpartum from both the mother and the newborn baby (the study sibling). Clinical evaluations are conducted four times, beginning at six months of age and concluding when the child is three years old. The evaluations include autism and behavioral assessments and dysmorphology examinations. At the final study visit when the child is 36 months of age, the sibling's ASD status is determined for all participants, although individual diagnoses may have been made earlier, depending on when symptoms emerged. Throughout participation in the study (prenatally and postdelivery) self-report data are collected from the mothers by using EARLI instruments and interviews that cover health behaviors, diet, reproductive and medical history, stress, depression, environmental and occupational exposures and medication use. Additional data are collected from the mother about the sibling during the first three years of life regarding general health, medications and medical care, specific symptoms or illnesses, diet, environmental exposures and developmental interventions. The EARLI Study is on pace to enroll 870 families over a 6-year period with plans in place to acquire follow-up data through 36 months from 630 of these families. Figure 1 shows key elements in EARLI Study data collection over the course of a family's participation

#### Study population

Women who meet the following criteria are eligible to participate in the EARLI Study: (1) have a biological child who has been diagnosed with an ASD, (2) competent to communicate in English (or, at two sites, in Spanish), (3) 18 years of age or older, (4) live within two hours of a study clinic and (5) are no more than 28 weeks pregnant. Women who meet the first four criteria and are not pregnant but trying to become pregnant or may become pregnant in the future (for example, unplanned pregnancy) may be followed and contacted regularly to ascertain their reproductive status. If they become pregnant during this preenrollment period, they can be rescreened for eligibility to enroll.

The EARLI Study is being implemented at four field sites in three distinct locations in the US, representing a racially, ethnically, and socioeconomically diverse study population (Table 1). The sites are based in major metropolitan areas (that is, Philadelphia, Baltimore, San Francisco Bay Area, and Sacramento) and catchment areas expand to a 2-hour radius of the study clinic at each site. Table 1 lists the range of county level demographics within the catchment areas.

**Table 1:** Range of the percentage of demographic characteristics among the counties in EARLI Study field site areas^a^

	County Low and High Percentages within Field Site Catchment Areas		
Characteristics	SE Pennsylvania (13 counties in PA, NJ, DE)	NE Maryland (9 counties in MD)	N. California (25 counties in CA)
White	41.0% to 89.2%	19.2% to 92.9%	43.0% to 91.4%
Black or African-American	3.6% to 43.4%	3.2% to 64.5%	0.4% to 14.7%
Asian	0.8% to 8.9%	1.1% to 14.4%	1.1% to 33.3%
Hispanic or Latino	3.0% to 18.8%	2.6% to 17.0%	8.5% to 55.4%
Language other than English spoken at home (5 years or older)^b^	7.4% to 24.8%	5.2% to 35.8%	6.5% to 51.2%
Families below poverty^c^	4.2% to 22.9%	4.5% to 22.9%	7.2% to 24.6%
25+ years old with educational attainment 9th to 12th grade, no diploma^†^	7.8% to 28.8%	5.7% to 31.6%	6.6% to 33.0%

^a^Data are derived from the 2010 US census. ^b^2005 to 2009 for all counties; 2000 for Philadelphia City and Baltimore City. ^c^2009 for all counties; 1999 for Philadelphia City and Baltimore City. EARLI, Early Autism Risk Longitudinal Investigation.

#### Participant recruitment

Recruitment strategies vary by field site to accommodate the unique resources available to each site. Generally, the target population is mothers with a young child (2 to 12 years old) with ASD, who would be more likely to become pregnant again than a mother with an older child. For example, for the Pennsylvania and Maryland sites, a primary strategy for reaching potentially eligible mothers is distribution of information through the early intervention and special education systems. The northern California site at the University of California, Davis, identifies and reaches potentially eligible mothers primarily through the state's Department of Developmental Services, whereas the Kaiser Permanente site in northern California can identify Kaiser Permanente members who become newly pregnant and already have children with autism. Clinical service providers in the catchment areas, including ASD evaluation and diagnostic centers, developmental pediatricians and mental health service providers, are also engaged at each site to reach potentially eligible women. The researchers in the EARLI Study have not focused on establishing relationships with providers serving the general population of pregnant women (for example, obstetricians, nurse midwives) or children (for example, general pediatricians) for individual-level outreach but will make available general information about the EARLI Study as requested. All field sites carry out supplementary recruitment efforts through staffed information tables at autism events and through advocacy organizations' websites, listservs and newsletters. Reinforcing information about the EARLI Study through multiple channels increases the chance that mothers of reproductive age who have children with ASD, at a later time when they are pregnant, will remember and consider enrolling in the EARLI Study. To that end, EARLI also maintains an active presence in social media, including Facebook [127] and YouTube [128] and has a web presence [129] that includes content geared toward potentially eligible mothers as well as enrolled participants. Given this recruitment approach, it is possible that participating families might differ from their respective area source population on factors related to the extent of connection to service systems and the degree of immersion in the autism community. To introduce bias, such selection needs to be differential with respect to both exposure and outcome. Although these selection effects could be associated with certain exposure profiles of interest, independent associations with ASD risk, though conceivable, seem less likely. EARLI Study sites, to varying degrees, will be able to explore differences between participating families and source populations. All sites can compare basic characteristics of participating families with families in the region receiving services for a young child with ASD, but only the Kaiser Permanente site has the ability to identify the subgroup in the source population who are becoming pregnant.

#### Enrollment and retention

When a pregnant mother of a child with autism begins the enrollment process, proband diagnosis first needs to be confirmed. In families with more than one child with ASD, the study proband is the child who is the closest biologic relation to the future sibling, or, if both are the same biologic relation to the future sibling, then a child with an autistic disorder diagnosis would be enrolled over a child with Asperger syndrome or PDD NOS. The mother is enrolled in the study after consenting at the enrollment clinic visit, and the study proband's eligibility is confirmed by a valid ADOS [109] and an age-appropriate IQ test (for example, Mullen Scales of Early Learning for infancy to 68 months of age; or the Kaufman Brief Intelligence Test, Second Edition, for ages 4 years to 90 years). Fathers may enroll at the enrollment clinic visit, or during a home visit if they are not at the enrollment clinic visit.

As Figure 1 illustrates, the EARLI Study involves extensive data collection, so investigators strive to maximize retention by being as flexible as possible. Both online and paper versions of most questionnaires and documents are available, and home visits and flexible visit scheduling is accommodated when possible. Of the first 177 families enrolled in the study, 97.2% are still participating. Variability in the time of data collection creates challenges and opportunities for research but is also a reality of intensive, prospective follow-up of this study population. Retention in the EARLI Study is also driven by the prospective developmental follow-up offered for the at-risk siblings. EARLI Study sites provide families with summaries of research evaluations, discuss questions with families, provide information on local resources for families concerned with their child's development and make referrals for services for affected siblings. Upon enrollment, the EARLI Study also provides families with a specially developed social storybook about the impending arrival of a baby sibling that parents can use in interactions with the proband. EARLI Study investigators stay connected with enrolled families as a group through the study website, Facebook and study newsletters.

#### Risk factor data collection

The EARLI Study approach to risk factor data collection is comprehensive, involving multimodal self-report, records review, direct observation and biologic and environmental sample collection. This approach allows for analysis of risk factor characterization during specific suspected etiologic windows, comparison of risk factor data from multiple sources, estimation of risk factor-outcome associations motivated by specific hypotheses and discovery-oriented work intended to reveal first evidence for novel risk factors. Table 2 provides a broad overview of data collection modes by subject. Self-reports are provided by both parents at enrollment and extend back to the preconception period. During pregnancy, mothers provide reports in weekly pregnancy diaries regarding exposures that are more challenging to recall retrospectively and are extensively interviewed twice (approximately 625 items) to collect information retrospectively on less time-sensitive information in pregnancy. Selected self-report questionnaires are also used to cover specific domains such as diet and depressive symptoms. Table 3 summarizes the range of risk factor domains covered by EARLI Study self-report data collection.

**Table 2:** EARLI Study data collection modes by subject^a^

	Mother					
Data collection mode	Preconception	Prenatal	Perinatal	Father	Proband	Sibling
Self-report retrospective	X	X		X	X	X
Self-report prospective		X				X
Biologic sampling		X	X	X	X	X
Direct observation (home environment)		X				X
Environmental sampling		X				X
Medical records^a^	X	X	X	X	X	X

^a^The EARLI Study secures medical record releases for each participant and will pursue abstraction as needed to support individual analyses. EARLI, Early Autism Risk Longitudinal Investigation.

**Table 3:** Domains of risk factor data collected from interviews, diaries and other self-report forms

Risk factor domains	Interviews	Diaries	Other self-report forms^a^
Demographics	M, F		F
Medication exposure	M, F	M, S	M, F, S
Medical conditions	M	M	M, F, S
Pesticides		S	M, S
Diet	M	S	M
Home environmental exposures	M	M, S	M, F
Health behaviors/lifestyle		S	M, F
Mental condition/history/symptoms	M	M	M, F, S
Vaccine history		M, S	
Personal product use	M	M, S	M
Anthropometrics	M	M	M
Medical procedures	M	M, S	M, F
Occupational history			M, F

^a^Includes the following forms: Home Walkthrough Survey, Maternal Interview Update, CHARGE Family Medical History Form, Dietary History Questionnaires (preconception, 1 to 20 weeks, 21 to 36 weeks and postnatal), Health Behaviors Questionnaires (preconception, pregnancy and paternal), Paternal Interview, 24-Hour Recalls (food and environment), Stress and Depression Surveys, Postnatal Diaries, Blood Draw Information Form, Maternal Medical History, Post-Partum Environmental Exposures and Dust Field Log. M, mother; F, father; S, sibling.

Biosampling in the EARLI Study is comparably extensive. Fathers and probands provide biosamples at enrollment. Venous blood is collected from both, and fathers are also provided with a home semen collection kit. Biosampling in mothers begins at enrollment with the collection of blood, first void urine and hair. Mothers provide these samples at least once and as many as three additional times during pregnancy, depending on how early in the pregnancy enrollment occurred. The EARLI Study makes efforts to work closely with mothers, obstetricians and/or birth hospitals to facilitate the collection of delivery samples. Umbilical cord blood and placental samples are collected as close to delivery as possible. Four placental punch biopsies, two from the maternal and two from the fetal side, are taken and placed into cryovials of RNA*later*™ (QIAGEN, Valencia, CA, USA). The remaining placental tissue is fixed in formalin. Heel stick cards are left at the hospital, and newborn blood is collected by hospital staff after neonatal screening whenever possible without an additional heel stick. Mothers are provided with collection kits for breast milk, meconium and diaper urine. Manually expressed breast milk is collected at one week (postcolostrum) and twelve weeks. Nighttime diaper urine is collected from a sterile gauze pad at one week. Study staff visit the family at three months and pick up biologic samples the family has retained in the home freezer and also collect clean-catch urine from the infant sibling at that time. At the six-month clinic visit, mothers again provide blood, urine and hair samples, and a first venous blood sample, another diaper urine sample and a hair sample are taken from the infant sibling. Biosampling concludes with infant siblings' providing venous blood and diaper/pull-up pad urine samples during the 12- and 24-month follow-up clinic visits. This continued longitudinal sampling of blood and urine in siblings provides opportunities for assessment of early life exposures and also creates the potential to investigate peripheral biomarkers of early outcome. Biosampling time points by participant and sample type are summarized in Table 4.

**Table 4:** EARLI biosampling time points by biosample and participant type^a^

Sample	Mother	Father	Proband	Infant sibling
Blood	E, pre-2nd, pre-3rd, post-6 months	E	E	Post-6 months, post-12 months, post-24 months
Hair	E, pre-2nd, pre-3rd, post-6 months			Post-6 months, post-12 months, post-24 months
Urine	E, pre-2nd, pre-3rd, post-6 months			Post-1 week, post-3 months, post-6 months, post-12 months, post-24 months
Semen		E		
Placenta, cord blood	D			
Heel stick blood, meconium				D
Breast milk	Post-1 week, post-3 months			

^a^D, delivery; E, enrollment; EARLI, Early Autism Risk Longitudinal Investigation; pre-2nd, second prenatal visit; pre-3rd, third prenatal visit; post-1 week, 1 week postnatal; post-3 months, 3 months postnatal; post-6 months, 6 months postnatal; post-12 months, 12 months postnatal; post-24 months, 24 months postnatal.

Biosample-processing decisions in EARLI were made by balancing timeliness, logistics and cost while being mindful of the nature of each sample. Processing on-site is minimal for venous blood, limited to centrifuging as dictated by tube type, with samples shipped next-day delivery to the central laboratory and biorepository (CLBR). The complement of tube types used varies slightly by subject and visit, but generally ethylenediaminetetraacetic acid (EDTA) and serum separator tubes (SSTs) are used during each draw with the PAXgene Blood RNA Kit (QIAGEN, Valencia, CA, USA), prescreened metal EDTA and cell preparation tubes (CPTs) interspersed. Maternal first void and infant sibling urine are aliquoted and frozen on-site and shipped monthly on dry ice to the CLBR. Diaper pad urine, meconium and breast milk are also batch-shipped frozen to the CLBR. Semen samples are collected by the father and frozen at home for a minimum of 24 hours, then shipped directly to the CLBR. Dried heel stick cards are sent back to the sites, where they are stored at ambient temperature and batch-shipped, as are hair samples, to the CLBR. Blood sample processing at the CLBR generates a repository of multiple aliquots of stored plasma, serum, whole blood, extracted DNA and peripheral blood mononuclear cells (PBMCs), including aliquots processed and saved to allow for establishment of cell lines.

Researchers in the EARLI Study also assess the home environment once during pregnancy and at the three-month postpartum home visit. The home assessment includes a walk-through survey with questions related to how the family distributes their indoor time across rooms in the home, characteristics of the principal rooms where the mother and infant sibling spend most of their time, cleaning product use, and indoor and outdoor spray and pesticide use. A dust sample is also collected from the main living area by using a Eureka Mighty Mite vacuum cleaner (Eureka Co, Charlotte, NC, USA) following a protocol used in multiple previous studies [130-134]. House dust is an easily collected reservoir comprising compounds such as pesticides, plasticizers and flame retardants and has served as a marker of exposure in several epidemiologic studies [135-139].

Finally, the members of the EARLI Study team obtain medical record release forms from all participants, and they plan to abstract records as needed to assess exposure related to clinical domains where self-report data have inherent limitations and/or where medical records data might validate recall. Items of particular interest include specific clinical tests or results in mothers (for example, type of ultrasound, blood pressure, blood glucose levels) and newborns (for example, oxygen saturation values, fetal heart rate tracings, newborn screening results) and details regarding indications and dates for procedures and medications.

#### Outcome data collection

Infant siblings are followed to age 36 months, with clinical assessment of ASD-related behaviors and other developmental domains occurring at 6, 12, 18, 24 and 36 months. Behavioral outcome assessment tools are summarized in Table 5. The assessment protocol was designed to measure core autism and related phenotypes, enabling investigation of dichotomous end points, continuous outcomes and developmental trajectories. Direct observation, interview and parent-report measures are all used. The autism-specific direct observation tool used at ages 6 and 12 months is the AOSI [115]. Initial evaluation of this tool suggested that total scores are the most robust predictor of autism at 24 months of age [115], and work is ongoing to assess the dimensional measure utility of the AOSI as well as the predictive ability of both total score and specific items [140]. At ages 24 and 36 months, the ADOS is administered. At these ages, the ADOS has high sensitivity for both autism and ASD, along with moderate specificity, using the revised scoring algorithm [141]. An algorithm has also recently been developed for converting raw ADOS scores to a 10-point severity measure (with scoring also dependent on ADOS module, classification and age) [142]. At 36 months, the ADI-R is also administered. The addition of the ADI-R to ADOS results for determining a final classification markedly improves classification specificity without major sacrifices in sensitivity [141]. The Social Responsiveness Scale (SRS) [143,144] has been shown to have useful dimensional scale properties in first-degree relatives of affected probands [145,146]. The EARLI researchers administer the Preschool Version (for 3-year-olds) of the SRS to infant siblings at age 36 months. At enrollment, the Adult Research Version of the SRS is administered to the parents, who each report on their spouse, and the Preschool Version or the Autoscore Form Parent Report is administered to the proband, depending on the proband's age.

**Table 5:** EARLI behavioral outcome assessments by infant sibling follow-up point^a^

Assessments	6-month clinic visit	12-month clinic visit	18-month mailing	24-month clinic visit	36-month clinic visit
Autism assessments					
AOSI (Autism Observation Scale for Infants)	X	X			
ADI-R (Autism Diagnostic Interview-Revised)					X
ADOS (Autism Diagnostic Observation Schedule)				X	X
SRS (Social Responsiveness Scale)					X
Other behavioral assessments					
CSBS-DP (Communication and Symbolic Behavior Scales Developmental Profile) Infant/Toddler Checklist	X	X	X		
CBCL (Child Behavior Checklist)					X
MCDI (MacArthur Communicative Development Inventories)		X	X	X	
M-CHAT(Modified Checklist for Autism in Toddlers)			X	X	
Mullen Scales of Early Learning	X	X		X	X
Rothbart Temperament Questionnaires	X				X
SEQ (Sensory Experiences Questionnaire)		X		X	X
Vineland II (Vineland Adaptive Behavior Scales, 2^nd ^edition)	X	X	X	X	X

^a^EARLI, Early Autism Risk Longitudinal Investigation.

In addition, EARLI incorporates other behavioral measures that have been demonstrated to have value in phenotyping high-risk infant siblings. The Mullen Scales of Early Leaning, in addition to providing data on subdomains of particular interest, such as nonverbal IQ [117] and motor functioning [147], has also been used more broadly to characterize developmental trajectory in high-risk siblings [10,148]. The Vineland Adaptive Behavior Scales [149,150] have been employed effectively with infant sibling data to differentiate functional phenotypes [148]. In addition, other tools can improve the richness of available data on early language and communication (Communication and Symbolic Behavior Scales Developmental Profile Infant/Toddler Checklist (CSBS DP ITC) [151,152] and MacArthur-Bates [153,154]), sensory impairments [155], temperament (Rothbart) [156,157] and emergent maladaptive behavioral and emotional problems (Child Behavior Checklist (CBCL) [158,159]).

The EARLI Study also incorporates data collection regarding physical features and medical comorbidities. At age 36 months, a dysmorphology assessment is completed following a protocol adapted from the Study to Explore Early Development (SEED) [160]. This examination involves direct measurement of growth parameters (height, weight, head circumference and body mass index) and evaluation of dysmorphology. A trained member of the research team photographs the child's face and ears (front of face, right and left profiles of ears, and left and right three-quarters images showing each ear and the face), hands (both sides and a hand scan of the palms), feet (weight-bearing and not), teeth, any skin findings and two posterior views of the head to identify hair whorl and hairline. For the facial photographs, a size reference sticker is included. Parents are queried about the presence of physical anomalies and whether the child has ever had corrective surgery, has been diagnosed with any syndromes or has had any genetic testing. This same assessment is administered to the proband at the time of enrollment. At the 6-, 12- and 24-month study visits, the sibling also receives a brief physical examination to capture infant growth parameters (length, weight, head circumference and weight-to-length ratio), and the parents are asked the same set of questions on genetic testing, anomaly or syndrome diagnoses and corrective surgeries. Finally, a comprehensive medical history questionnaire completed by the mother addresses any medical problems and procedures that have occurred during the course of the infant sibling's first three years of life. As mentioned above, medical records releases are obtained for the infant sibling to allow follow-up for more details on any problems or procedures noted.

The design, recruitment strategy and data collection approach of the EARLI Study are intended to build a data platform upon which a wide range of prenatal and early life risk factor investigations will be launched. The rich combination of prospectively collected exposure and outcome data should allow for analyses that incorporate strong confounder control and limit exposure misclassification and have a range of data sufficient to approach complex questions of effect modification and mediation along risk pathways. Although EARLI's sample size is large in relation to other infant sibling studies, there will no doubt be challenges related to sample size, and, as mentioned previously, attention to designing analytic contrasts in ways that maximize efficiency and incorporation of dimensional as well as categorical outcomes will likely prove helpful in this regard.

## Conclusions

Infant sibling studies have already played a major role in autism research over the past decade, improving our understanding of the complex early developmental trajectory of autism, providing exciting leads on approaches for early detection and documenting recurrence risk under today's diagnostic standards. Extension of the infant siblings design to intervention studies is already underway, with behavioral interventions being tested in high-risk siblings with very early signs of developmental issues (see, for example, the Infant Start Study [161]).

As described above, the potential for the extension of the design to autism risk factor research is great. The EARLI Study has substantial potential to contribute to risk factor research on its own; however, there is also added potential through collaborations and extensions of the EARLI project. The EARLI Study team is working with researchers in the Infant Brain Imaging Study (IBIS) [162], another extension of the infant siblings design adding prospective brain imaging to developmental follow-up, to conduct coordinated genomics on both EARLI and IBIS study samples to undertake pooled analyses of genetic variants and developmental phenotypes. Because both IBIS and EARLI are collecting phenotype data on infant siblings longitudinally from ages 6 to 36 months, they have a unique opportunity to examine genetic relationships with developmental trajectories in addition to autism *per se*. Moreover, the genetic data will support independent analyses of genotypes and brain imaging in the IBIS sample and gene-environment interaction in EARLI. EARLI has also partnered with experts in epigenetics to explore the potential role of epigenetic mechanisms in autism and the possible link between epigenetics and environmental risk factors. Through a National Institutes of Health Roadmap program award, EARLI data will be analyzed in parallel with data from two other birth cohort studies to examine relationships between DNA methylation (DNAm) and prenatal exposures, as well as between DNAm, birth outcomes and early childhood developmental milestones. As the EARLI cohort develops, other opportunities to take advantage of the rich available data in this unique sample are sure to arise.

Last, in addition to enriched risk pregnancy cohorts such as EARLI, it should be noted that worldwide there are several population-based pregnancy cohort studies, in which recruitment is not geared to enriched-risk families, that have made autism an identified outcome of interest [163-167]. These studies range in size from the 1,200-subject Hamamatsu Birth Cohort in Japan [168] to the 110,000-subject Autism Birth Cohort Study [167] that has recently been incorporated into the Norwegian Mothers and Babies Study [169]. Researchers in population-based cohort studies can explore the generalizability of findings that emerge from enriched-risk designs and could also become engaged with enriched-risk cohorts in coordinated analytic efforts to study rare prenatal exposures or complex etiologic mechanisms.

As research on autism risk factors and risk biomarkers during the pre-, peri- and neonatal periods intensifies during the coming decade, enriched-risk cohort designs, along with large case-control studies [170,171] and population-based cohort designs, can be expected to play an important role. This expansion of autism infant sibling studies, which have emerged during the past five years as extremely valuable tools with which to improve understanding of the early-life autism phenotype, to address etiologic questions will, we hope, mark an important step toward identification of avoidable or modifiable factors that will ultimately help reduce the population morbidity and impact of autism on quality of life.

## Abbreviations

ADI-R: Autism Diagnostic Interview-Revised; ADOS: Autism Diagnostic Observation Schedule; AOSI: Autism Observation Scale for Infants; ASD: Autism spectrum disorder; BSRC: Baby Siblings Research Consortium; CBCL: Child Behavior Checklist; CLBR: Central laboratory and biorepository; CNV: Copy number variation; CPT: Cell preparation tube; CSBS DP: Communication and Symbolic Behavior Scales Developmental Profile; DNAm: DNA methylation; EARLI: Early Autism Risk Longitudinal Investigation; EDTA: Ethylenediaminetetraacetic acid; GWAS: Genomewide association study; IBIS: Infant Brain Imaging Study; MARBLES: Markers Of Autism Risk In Babies-Learning Early Signs; PBMC: Peripheral blood mononuclear cell; PDD NOS: Pervasive developmental disorder not otherwise specified; SEED: Study to Explore Early Development; SST: Serum separator tube; SRS: Social Responsiveness Scale.

## Competing interests

The authors declare that they have no competing interests.

## Authors' contributions

CJN, LAC, MDF, and IHP made substantial contributions to the conception and design of the study, and drafted and revised the manuscript. DVN, SMM, and AS participated in data coordination, collection, and analysis, and revised the manuscript. NLL, CAB, MLM, MCO, and KMSW contributed to the implementation of the study and contributed to manuscript revisions. HF and SCM participated in the design and coordination of biosampling aspects of study implementation, and revised the manuscript. HNH, SEL, RJL, SO, and JP contributed to the study design and clinical data collection, and contributed to the manuscript draft and revisions. All authors read and approved the final manuscript.
